# Impact of the Tranexamic Acid on Bleeding Amount of Surgical Patient With Degenerative Spinal Disease: A Randomized Blinded Study

**DOI:** 10.3389/fsurg.2021.655692

**Published:** 2021-10-26

**Authors:** Lei Yan, Huihong Yang, Haibin Jiang, Mingshan Yu, Jie Tan, Tao Su, Guiping Xu

**Affiliations:** ^1^Department of Anesthesiology, People's Hospital of Xinjiang Uygur Autonomous Region, Urumqi, China; ^2^Medical School, Shihezi University, Xinjiang Uygur Autonomous Region, Shihezi, China; ^3^Xinjiang Uygur Autonomous Region, Xinjiang Medical University, Urumqi, China

**Keywords:** tranexamic acid, spinal degenerative disease, bleeding volume, TXA, postoperative rehabilitation

## Abstract

**Objective:** This study aims to explore the effectiveness and safety of tranexamic acid (TXA) in reducing the bleeding amount of surgical patients with degenerative spinal disease in the perioperative period.

**Methods:** A total of 80 cases of patients, who underwent elective posterior lumbar interbody fusion surgeries under general anesthesia, were enrolled in this study. The age of these patients ranged within 41–69 years old, and the surgical vertebral body segments were ≥2. The ASA classification was Level I or Level II. These patients were divided into two groups using the random number table (*n* = 40): TXA group and control group (S group). In the TXA group, the skin was incised after the anesthesia induction, and 20 mg/kg of TXA was immediately injected into the vein. The injection continued at a rate of 10 mg·kg^−1^·h^−1^ during the surgery, until the surgery was finished. In the S group, IV and pump injection with an equal amount of normal saline (NS) were performed. Then, the RBC, Hb, HCT, AST, ALT, BUN, Cr, PT, TT, APTT, FIB, and D-dimer were measured before the surgery and at 1 day after the surgery, and the SSFQ, intraoperative bleeding amount, homologous transfusion volume, urine volume, infusion quantity, surgical duration, drainage volume at 24 h after the surgery, total bleeding amount and adverse event occurrence at 1 week after the surgery were recorded.

**Results:** The RBC, Hb and HCT at 1 day after the surgery were higher in TXA group than in the S group (average *P* < 0.05). Intraoperative bleeding, drainage volume at 24 h after surgery, and total blood loss were lower in the TXA group than in the S group (average *P* < 0.05). The SSFQ score and length of stay were lesser in the TXA group than in the S group (average *P* < 0.05). The differences in AST, ALT, BUN, Cr, PT, TT, APTT, FIB, and D-dimer at 1 day after the surgery for these two groups of patients had no statistical significance (average *P* > 0.05).

**Conclusion:** TXA can reduce the bleeding amount of surgical patients with degenerative spinal disease in the perioperative period and decrease the length of stay, but does not increase the occurrence rate of adverse events, thereby promoting postoperative rehabilitation.

**Clinical Trial Registration:** www.chictr.org.cn/index.aspx, identifier: ChiCTR2000033597.

## Introduction

The common causes of spinal disease can be divided into four categories: trauma, tumor, deformity, and degenerative disease. The differences in spinal diseases may result in different bleeding amounts during the perioperative period. The three principles that undergo spinal disease surgery are pressure reduction, fixation and fusion. For patients with simple spinal instability, the purpose of surgery is to rebuild the stability of the spine. For patients accompanied with spinal nerve damage, the purpose of the surgery is to decompress the spinal nerve and rebuild the stability of the spine.

The bleeding amount during the spinal perioperative period is impacted by the type of spinal disease and spinal surgery method. With the growing aging population, the morbidity of chronic degenerative diseases continuous to increase, and the rate of spinal surgery is also increasing (1). Spinal degenerative disease refers to the spinal intervertebral disc degeneration, and its secondary pathologic change involves the surrounding tissue structures, which leads to the corresponding clinical manifestation. With the growing number of surgical patients with degenerative spinal disease in the perioperative period, overall blood conservation strategies are crucial for reducing the occurrence of complication in the perioperative period, and improving the overall effect of the surgical treatment (2). At present, blood conservation strategies during the perioperative period include the following: preoperative autologous blood donation, acute normovolemic hemodilution, autologous blood transfusion, controlled hypotension, and the use of antiplasmin drugs (3). In the clinical use of antiplasmin drugs, TXA has been widely used during the perioperative period. TXA is a lysine analog and an antifibrinolytic agent. It reversibly inhibits the activation of plasmin by plasminogen by binding to a specific lysine site of plasminogen, thereby delaying blood. The clot degrades and is widely used to reduce perioperative blood loss (4). TXA has been used in cardiac and orthopedic surgeries, including major spinal surgeries, to reduce blood loss and transfusion requirements for decades (5). TXA may reduce blood transfusion requirements and postoperative complications following spinal procedures. Studies investigating the role of TXA in spine surgery have presented promising results and have proven its safety and efficacy. However, further investigation is needed to determine the optimal dosing regimen of TXA (6–10). This study was designed to investigate the hypothesis that TXA reduces perioperative blood loss and transfusion requirements in patients undergoing degenerative spinal disease.

## Methods

### Object of Research

This controlled trial was approved by the Ethics Committee of People's Hospital of Xinjiang Uygur Autonomous Region, and registered in the Chinese Clinical Trial Register website (http://www.chictr.org.cn/index.aspx, ChiCTR2000033597, 2020-06-06). Furthermore, all patients or relatives were informed of the study, and provided a signed informed consent. The patients with degenerative spinal disease, who received treatment in Xinjiang Uiger Municipal People's Hospital, were divided into two groups: TXA group and control group (S group). A table of random numbers hidden in the 1:1 ratio was computer-formed. A researcher who did not take part in the trial used the website Randomization.com to generate a random distribution sequence, which was hidden in sealed opaque sequence numbered envelopes that were allocated to investigators. The patients were all kept blinded to allocation results.

Inclusion criteria: (1) patients >40 years old, with no gender limitation, and patients with an American Society of Anesthesiologists (ASA) classification of Level I or Level II; (2) patients with no allergic history of opioid drugs and TXA; (3) patients with no oral administration of anticoagulant drugs or antiplatelet drugs; (4) patient with no coagulation disorders; (5) patients who were willing to cooperate with the investigation and provide a signed informed consent.

Exclusion criteria: (1) patients who could not normally communicate or refuse to cooperate; (2) patients who had subarachnoid space bleeding; (3) patients who had venous thrombus before surgery; (4) patients accompanied by vitals complication; (5) patients with blindness or acquired visual impairment.

### Research Method

The fasting for solids and liquids should be subject to the preoperative fasting guide. The routine monitoring of heart rate, blood pressure, electrocardiogram (ECG), oxyhemoglobin saturation, and depth of anesthesia was conducted after the patients entered the room. The venous channel of the upper limb was opened, left radial artery puncture and cannulation were performed under the local anesthesia, the hemodynamic indexes were monitored, and the blood gas was measured. Conventional anesthesia induction and maintenance was performed, maintain the BIS value within 45–60. Mechanical ventilation was performed after endotracheal intubation, in order to maintain the end-expiratory partial pressure of carbon dioxide of 35–45 mmHg. The right internal jugular venous puncture and cannulation were performed for infusion and blood transfusion, as well as the measurement of central venous pressure (CVP) and SVO_2_.

After the skin incision, in the TXA group, 20 mg/kg of TXA was immediately injected into the vein. The injection was continued at a rate of 10 mg·kg^−1^·h^−1^ during surgery, until the surgery was finished. In the S group, IV administration and pump injection with an equal amount of normal saline (NS) were performed.

Primary research endpoint were intraoperative bleeding amount, homologous transfusion volume, and the scores of surgical field quality (SSFQ). And secondary research endpoint were Liver and kidney, coagulation function and adverse event occurrence.

The scores of surgical field quality (SSFQ) uses 1–5 points to evaluate the surgical field. 1 point: slight bleeding, no attraction required; 2 points: slight bleeding, occasional attraction, without obstructing the surgical field; 3 points: slight bleeding, frequent aspiration, bleeding hindered the surgical field a few seconds after stopping the suction; 4 points: moderate bleeding, Bleeding requires frequent suction. Bleeding after stopping aspiration will immediately hinder the surgical field; 5 min of severe bleeding requires continuous suction, and bleeding suction still hinders the surgical field. The blood transfusion was conducted according to the intraoperative blood gas measurement indexes, when Hb was <70 g/L or Hct was <25%.

The multi-functional monitor was used to dynamically monitor the changes in the indexes of the patient, such as HR, MAP, SpO2, CVP, and BIS. In addition, the blood gas analysis was carried out in a time manner: Hb, HCT, lactic acid level, and SVO2. The fluid infusion and blood transfusion were guided to maintain the homeostasis, and guarantee the tissue perfusion. The tracheal catheter was pulled out after the patient had spontaneous breath and recovered consciousness after surgery. Then, the patient was sent to the anesthesia recovery room.

### Quality Control

All surgical operations were performed by surgeons from the same group, in order to avoid the influence of different operation technologies on the bleeding amount. The research object and researcher did not know about the experimental groupings and the drugs for the pump injection given to the researcher before the anesthesia induction was performed by the experimental design personnel. During the whole experimental process, the experimental design personnel did not participate in the assessment of the patient's condition, bleeding amount, and the scores of surgical field quality (SSFQ).

### Data Collection

The basic information of the patient, such as age, gender, height and weight, were collected. Furthermore, the following were recorded: red blood cells (RBC), hemoglobin (Hb), hematocrit (HCT), aspartate amino transferase (AST), alanine aminotransferase (ALT), blood urea nitrogen (BUN), creatinine (Cr), prothrombin time (PT), thrombin time (TT), activated partial thromboplastin time (APTT), fibrinogen (FIB), and D-dimer at 1 day before surgery (T1) and after surgery (T2), and SSFQ; intraoperative blood loss, blood transfusion volume, urine volume, surgical duration, drainage volume at 24 h after surgery and length of stay; the adverse event occurrence of headache, nausea, emesis, diarrhea, epidural hematoma, deep venous thrombosis, pulmonary embolism, allergic reaction, epilepsy, heart cerebrovascular accident, and abnormal renal and liver function at 1 week after the surgery. The data collectors for the present study underwent unified training before the research and investigation, and the experimental design personnel did not take part in the data collection. Specially assigned individuals were allocated to review the investigation results. These pairs checked the accuracy of the data entry after entering all original data into Excel on the same day.

### Sample Size Calculation

The sample size was calculated based on the formula: n = 2 × [(μ_α_-μ_β_)σ/δ], where α = 0.05, 1-β = 0.90. According to our preliminary study, the mean blood loss of control group was 483 ml, while the mean blood loss of TXA group was 314 ml. The σ was 188. PASS was used to calculated the sample size. For each group, 27 patients was enough to get the power of 90%. Considering the expulsion cases, we enrolled 40 patients for each group.

### Statistical Analysis

The data analysis was conducted using the SPSS 20.0 statistical software. Normally distributed measurement data were expressed in x ± standard deviation (SD), and the comparison between groups was performed using independent sample *t*-test. Normally distributed measurement data were presented in M (P25, P75), and the comparison among groups was performed using the rank sum test. Measurement data were expressed in x ± SD, and *X*^2^-test was used for comparisons between groups. *P* < 0.05 was considered statistically significant.

## Results

### Comparison of the General Information of These Two Groups of Patients

**Figure 1:**
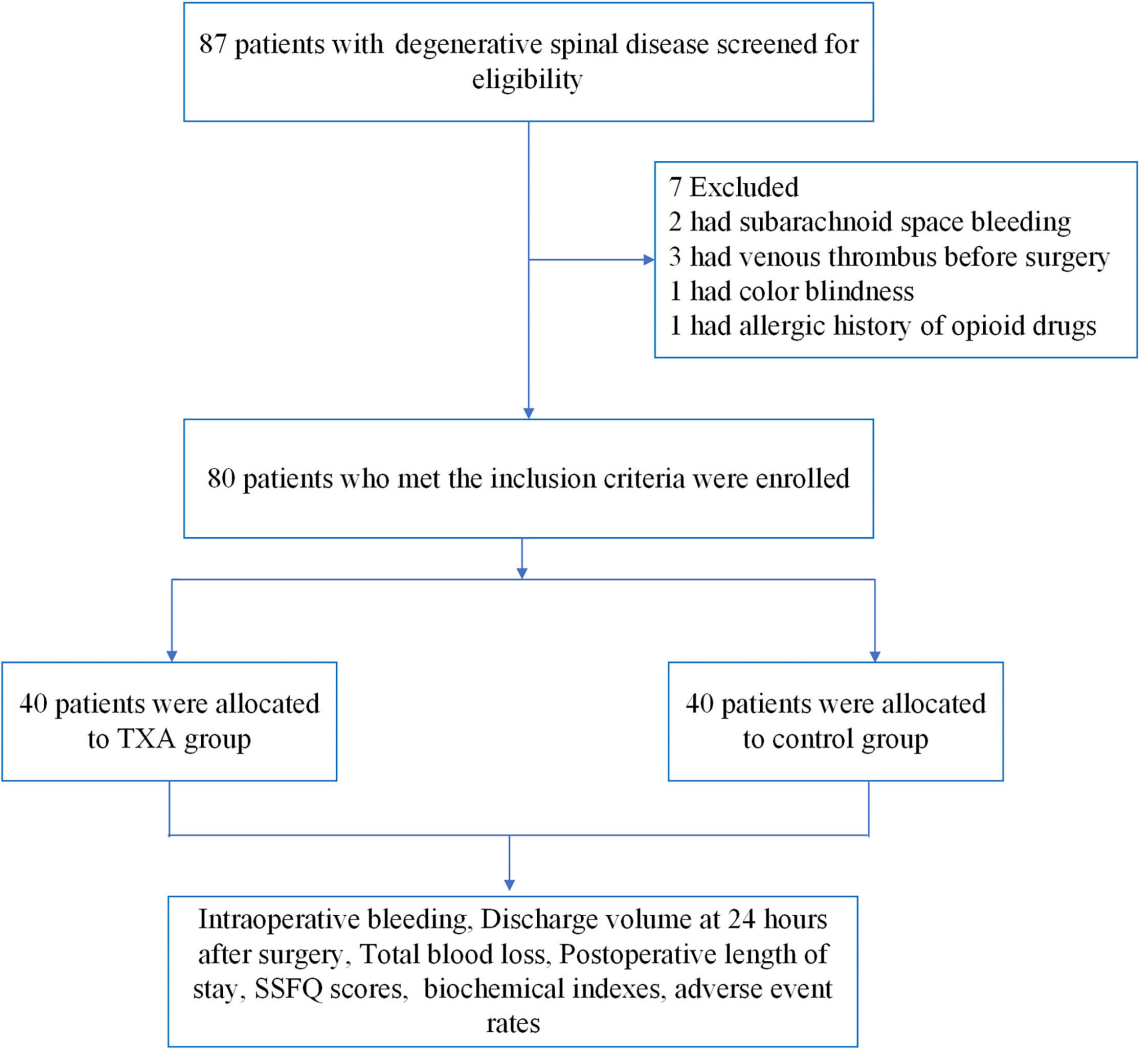
The flow chart of this study.

From April 2018 to July 2019, a total of 87 patients with degenerative spinal disease were screened, and 80 patients were randomly assigned to trial treatment (40 to S group and 40 to TXA group, Figure 1). The age, weight, body mass index (BMI) and gender of these two groups differed, but the difference was not statistically significant (*P* > 0.05, Table 1).

**Table 1:** Comparison of the general information of these two groups of patients ($\bar{x}$ ± s).

**Groups**	**Number of cases**	**Age**	**Weight (kg)**	**BMI**	**Gender (male/female)**
S group TXA group	40 40	52 ± 8 53 ± 7	67 ± 12 70 ± 10	24.4 ± 3.4 25.4 ± 3.1	22/18 16/24
t/χ^2^ value *P*		−0.213 0.832	−0.921 0.360	−1.482 0.142	1.805 0.179

### Comparison of Surgery-Related Data Between the Two Groups of Patients

The differences in surgical duration, infusion quantity and urine volume between these two groups were not statistically significant (*P* > 0.05). In the TXA group, the intraoperative bleeding, discharge volume at 24 h after surgery, total blood loss, postoperative length of stay and SSFQ scores were significantly lower, when compared to the S group, and the differences were statistically significant (*P* < 0.05, Table 2).

**Table 2:** Comparison of surgery-related data between these two groups of patients ($\bar{x}$ ± s).

**Groups**	**Number of cases**	**Surgical duration (min)**	**Infusion quantity (mL)**	**Urine volume (mL)**	**Intraoperative bleeding (mL)**
S group	40	187 ± 24	2 195 ± 223	411 ± 118	397 ± 90
TXA group	40	183 ± 21	2 103 ± 218	406 ± 116	240 ± 56^a^
*T* value		0.708	1.877	0.172	9.355
*P* value		0.481	0.064	0.864	0.000
**Groups**	**Number of cases**	**Discharge volume at 24 h after surgery (mL)**	**Total blood loss (mL)**	**Postoperative length of stay (d)**	**SSFQ (scores)**
S group	40	108 ± 34	505 ± 101	8 ± 3	2.58 ± 0.55
TXA group	40	65 ± 25^a^	304 ± 66^a^	6 ± 2^a^	2.23 ± 0.42^a^
*T*		6.469	10.543	3.077	3.192
*P*		0.000	0.000	0.003	0.002

*Compared with S group*.

a *P < 0.05*.

### Comparison of Biochemical Indexes Between the Two Groups of Patients Before Surgery and at 1 Day After Surgery

The differences in preoperative biochemical indexes between these two groups of patients were not statistically significant (*P* > 0.05). The RBC, Hb and HCT at 1 day after the surgery were apparently higher in the TXA group than in the S group, and the difference was statistically significant (*P* < 0.05). However, the differences in the other biochemical indexes at 1 day after the surgery was not statistically significant (*P* > 0.05, Table 3).

**Table 3:** Comparison of biochemical indexes between these two groups of patients before surgery and at 1 day after surgery ($\bar{x}$ ± s).

**Groups**	**Number of cases**	**RBC (×10** ^**9**^ **/L)**		**HB (g/L)**		**HCT (%)**	
		**T1**	**T2**	**T1**	**T2**	**T1**	**T2**
S group	40	4.6 ± 0.5	4.0 ± 0.5	139 ± 14	122 ± 16	41 ± 4	36 ± 4
TXA group	40	4.6 ± 0.5	4.3 ± 0.5^a^	140 ± 15	130 ± 15^a^	42 ± 4	38 ± 4^a^
*T* value		0.447	0.599	−0.489	−2.405	−0.366	−2.516
*P* value		0.718	0.004	0.627	0.019	0.716	0.014
**Groups**	**Number of cases**	**AST (U/L)**		**BUN (mmol/L)**		**Cr (μmol/L)**	
		**T1**	**T2**	**T1**	**T2**	**T1**	**T2**
S group	40	18 ± 6	22 ± 7	4.9 ± 1.4	5.6 ± 1.9	59 ± 13	59 ± 13
TXA group	40	19 ± 5	24 ± 9	4.5 ± 1.6	5.0 ± 1.4	60 ± 11	60 ± 14
*T* value		−0.510	−1.183	1.195	1.681	−0.182	−0.260
*P* value		0.612	0.240	0.236	0.097	0.856	0.795
**Groups**	**Number of cases**	**TT (s)**		**APTT (s)**		**D-dimer (μg/L)**	
		**T1**	**T2**	**T1**	**T2**	**T1**	**T2**
S group	40	18.1 ± 1.4	20.4 ± 1.5	29.0 ± 3.6	33.6 ± 4.3	0.38 ± 0.18	0.46 ± 0.18
TXA group	40	18.1 ± 1.6	20.0 ± 1.5	28.6 ± 2.9	32.7 ± 3.9	0.36 ± 0.19	0.45 ± 0.19
*T* value		0.186	1.511	0.487	0.933	0.355	0.155
*P* value		0.853	0.135	0.627	0.354	0.723	0.877

*Compared with S group*.

a *P < 0.05*.

### Comparison of Intraoperative Blood Transfusion Rates and Adverse Event Rates at 1 Day After the Surgery Between the Two Groups of Patients

The differences in intraoperative blood transfusion rates between these two groups of patients was not statistically significant (*P* > 0.05). Furthermore, these two groups of patients did not have adverse events within 1 week after surgery, such as headache, nausea, emesis, diarrhea, epidural hematoma, deep venous thrombosis, pulmonary embolism, allergic reaction, epilepsy, heart cerebrovascular accident, abnormal renal, and liver function, etc. (Table 4).

**Table 4:** Comparisons of intraoperative blood transfusion rates and adverse event rates at 1 day after surgery between these two groups of patients (%).

**Groups**	**Number of cases**	**Blood transfusion event**		**Blood transfusion rates (%)**	**Adverse event**		**Event rates (%)**
		**Transfusion**	**No transfusion**		**Yes**	**NO**	
S group	40	3	37	7.5	0	40	0
TXA group	40	1	39	2.5	0	40	0
χ^2^ value				0.263			
*P* value				0.608			

## Discussion

In the present study, we found that TXA could reduce the bleeding amount of surgical patients with degenerative spinal disease in the perioperative period and decrease the length of stay, thereby promoting postoperative rehabilitation.

In the assessment of the effectiveness of TXA in reducing the bleeding amount during spinal degenerative disease surgery, our results revealed that intraoperative bleeding, drainage volume at 24 h after surgery, and the total bleeding amount were significantly lower in the TXA group than in the S group. Furthermore, the RBC Hb and HCT at 1 day after the surgery were apparently higher in the TXA group than in the S group. These results were consistent with a study on the reduction of the curative effect of instable degenerative spinal canal stenosis bleeding by TXA during posterior lumbar surgery, which was conducted by Endres et al. (11). This indicates that TXA can reduce perioperative blood loss during spinal degenerative disease surgery. The present study results revealed that the IV administration of can reduce the SSFQ, which was conducive in allowing the surgeon to more clearly see the surgical field during the surgical operation, thereby reducing the bleeding during the operation. The length of stay after surgery was significantly lower in patients in the TXA group than in patients in the S group. This was more conducive for discharging the patient from the hospital, reducing the incidence of acquiring infection in the hospital and medical costs, and promoting postoperative rehabilitation.

The present study compared the ALT, AST, BUN, Cr, PT, TT, APTT, FIB, and D-dimer levels before surgery and at 1 day after surgery between these two groups of patients, in terms of the safety assessment of TXA in reducing blood loss during spinal degenerative disease surgery. The results revealed that the differences in these indexes between these two groups of patients was not statistically significant (*P* > 0.05). Furthermore, these two groups of patients did not have adverse events within 1 week after surgery, such as headache, nausea, emesis, diarrhea, epidural hematoma, deep venous thrombosis, pulmonary embolism, allergic reaction, epilepsy, heart cerebrovascular accident, etc. These results have proven that the IV infusion of TXA during spinal degenerative disease surgery has good safety and effectiveness.

In the present study, merely one patient from the TXA group during the perioperative period was infused with allogeneic blood, while three patients from the S group were infused with this, but the comparative differences in blood transfusion rate was not statistically significant (*P* > 0.05). These results reveal that the IV infusion of TXA did not reduce the blood transfusion rate during the perioperative period, but significantly decreased the perioperative blood loss of patients with spinal degenerative disease. This is consistent with the use of TXA in the spinal surgery of adults in a study conducted by Colomina et al. (12). The study reported that TXA could reduce the intraoperative allogeneic blood infusion rate when cutting down the bleeding amount during the spinal surgery. Cheriyan and Maier et al. have proven in many analysis reports that the average reduction in intraoperative, postoperative and total bleeding amount in the TXA group was 219, 119, and 201 mL, respectively, and that the blood transfusion rate decreases by 33%, when compared to the S group (4). However, the present study failed to draw a conclusion for determining whether the IV administration of TXA during spinal degenerative disease surgery could decrease the allogeneic blood infusion rate. Hence, further research is necessary.

There were certain limitations in the present study. (1) The research objects included into the present study were relatively few, which may have caused the adverse reactions not to appear after the IV infusion of TXA. Therefore, further multicenter, large-scale and double-blind randomized controlled trials are required. (2) For adverse events during the follow-up visits, the present study followed up the occurrence rate of adverse events at 1 day after surgery. In the future, follow-up visits for adverse events at 1 month, and at an even longer time after surgery would be further conducted, in order to prove the long-term safety of TXA. (3) For determining whether the IV administration of TXA during spinal degenerative disease surgery can decrease the allogeneic blood infusion rate, the present study failed to draw a conclusion. This may be due to the few number of research objects. Hence, further research is necessary.

## Conclusion

This study disclosed that although blood resources should be preserved for resolving blood shortage issues clinically, TXA could reduce the bleeding amount of surgical patients with degenerative spinal disease in the perioperative period and decrease the length of stay, but did not increase the occurrence rate of adverse events, thereby promoting postoperative rehabilitation.

## Data Availability Statement

The original contributions presented in the study are included in the article/supplementary material, further inquiries can be directed to the corresponding author/s.

## Ethics Statement

The studies involving human participants were reviewed and approved by the Ethics Committee of People's Hospital of Xinjiang Uygur Autonomous Region, and registered in the Chinese Clinical Trial Register website (http://www.chictr.org.cn/index.aspx, ChiCTR2000033597, 2020-06-06). The patients/participants provided their written informed consent to participate in this study.

## Author Contributions

LY and HY: conception and design of the research. HJ and TS: acquisition of data. MY: analysis and interpretation of the data. JT: statistical analysis and writing of the manuscript. GX: obtaining financing. GX and LY: critical revision of the manuscript for intellectual content. All authors contributed to the article and approved the submitted version.

## Funding

Natural Science Foundation of Xinjiang Uygur Autonomous Region (2017D01C137).

## Conflict of Interest

The authors declare that the research was conducted in the absence of any commercial or financial relationships that could be construed as a potential conflict of interest.

## Publisher's Note

All claims expressed in this article are solely those of the authors and do not necessarily represent those of their affiliated organizations, or those of the publisher, the editors and the reviewers. Any product that may be evaluated in this article, or claim that may be made by its manufacturer, is not guaranteed or endorsed by the publisher.
